# Antibodies with ‘Original Antigenic Sin’ Properties Are Valuable Components of Secondary Immune Responses to Influenza Viruses

**DOI:** 10.1371/journal.ppat.1005806

**Published:** 2016-08-18

**Authors:** Susanne L. Linderman, Scott E. Hensley

**Affiliations:** 1 Department of Microbiology, Perelman School of Medicine, The University of Pennsylvania, Philadelphia, Pennsylvania, United States of America; 2 Wistar Institute, Philadelphia, Pennsylvania, United States of America; University of Rochester Medical Center, UNITED STATES

## Abstract

Human antibodies (Abs) elicited by influenza viruses often bind with a high affinity to past influenza virus strains, but paradoxically, do not bind to the viral strain actually eliciting the response. This phenomena is called ‘original antigenic sin’ (OAS) since this can occur at the expense of generating new *de novo* Abs. Here, we characterized the specificity and functionality of Abs elicited in mice that were sequentially exposed to two antigenically distinct H1N1 influenza virus strains. Many Abs elicited under these conditions had an OAS phenotype, in that they bound strongly to the viral strain used for the first exposure and very weakly to the viral strain used for the second exposure. We found that OAS and non-OAS Abs target the same general region of the influenza hemagglutinin protein and that B cells expressing these two types of Abs can be clonally-related. Surprisingly, although OAS Abs bound with very low affinities, some were able to effectively protect against an antigenically drifted viral strain following passive transfer *in vivo*. Taken together, our data indicate that OAS Abs share some level of cross-reactivity between priming and recall viral strains and that B cells producing these Abs can be protective when recalled into secondary immune responses.

## Author Summary

Humans are continuously exposed to antigenically distinct influenza virus strains. Influenza virus infections early in life elicit immune responses that are subsequently recalled upon exposures with different influenza virus strains. Antibodies elicited against older influenza strains can dominate immune responses elicited against new influenza strains. This process is referred to as ‘original antigenic sin’ since the recall of antibodies against past influenza strains can occur at the apparent expense of generating antibodies against new viral strains. It has been proposed that ‘original antigenic sin’ contributes to vaccine failures. Here, we use a mouse model to show that antibodies that have an ‘original antigenic sin’ phenotype surprisingly target the same regions of influenza viruses that are recognized by strain-specific antibodies. Most importantly, despite the observation that ‘original antigenic sin’ antibodies bound poorly in conventional binding assays, these antibodies were able to efficiently protect *in vivo*. These data indicate that antibodies with an ‘original antigenic sin’ phenotype are an underappreciated valuable component of secondary immune responses to influenza viruses.

## Introduction

Influenza viruses continuously acquire mutations in antigenically important regions of the hemagglutinin (HA) and neuraminidase (NA) proteins through a process termed ‘antigenic drift’. Single HA mutations can dramatically reduce antibody (Ab) recognition of influenza viruses [1]. Although influenza infections lead to strain-specific lifelong immunity [2], humans are routinely re-infected with antigenically drifted influenza strains throughout life. Most humans are infected with seasonal influenza viruses by 3 years of age [3] and then re-infected with antigenically drifted strains every 5–10 years [4].

Influenza infections early in life can leave long-lived immunological imprints. In 1960, Thomas Francis coined the term ‘original antigenic sin (OAS)’ to describe the observation that Abs primed by older viral strains often dominate secondary responses elicited by new antigenically drifted viral strains [5]. The ‘S’ in OAS refers to the observation that the recall of Abs generated against past strains can occur at the apparent expense of generating *de novo* Abs that react to new viral strains [5,6]. It is thought that OAS Abs are detrimental to the host since they react poorly to the viral strain that is actually recalling them.

While it is clear that the majority of human influenza infections take place in the context of a previously exposed host, most animal models do not take prior-exposures into account. Recently, Jacobs and colleagues established a mouse model of OAS [7,8]. These studies show that OAS Abs can be elicited in mice following sequential exposures with antigenically distinct influenza strains. Here, we used a similar mouse model to determine the precise binding footprints and neutralization efficiencies of OAS Abs elicited by sequential influenza exposures. Surprisingly, we found that many OAS Abs target the same general region of HA that is recognized by non-OAS Abs. Further, we found that OAS Abs can be highly effective at neutralizing antigenically distinct viruses *in vivo* and that B cells producing OAS Abs are clonally related to B cells that produce non-OAS Abs. Taken together, our data suggest that Abs with an OAS phenotype are a valuable protective component of secondary influenza immune responses.

## Results

### Establishment of a mouse model of ‘OAS’ Ab production

**Fig 1:**
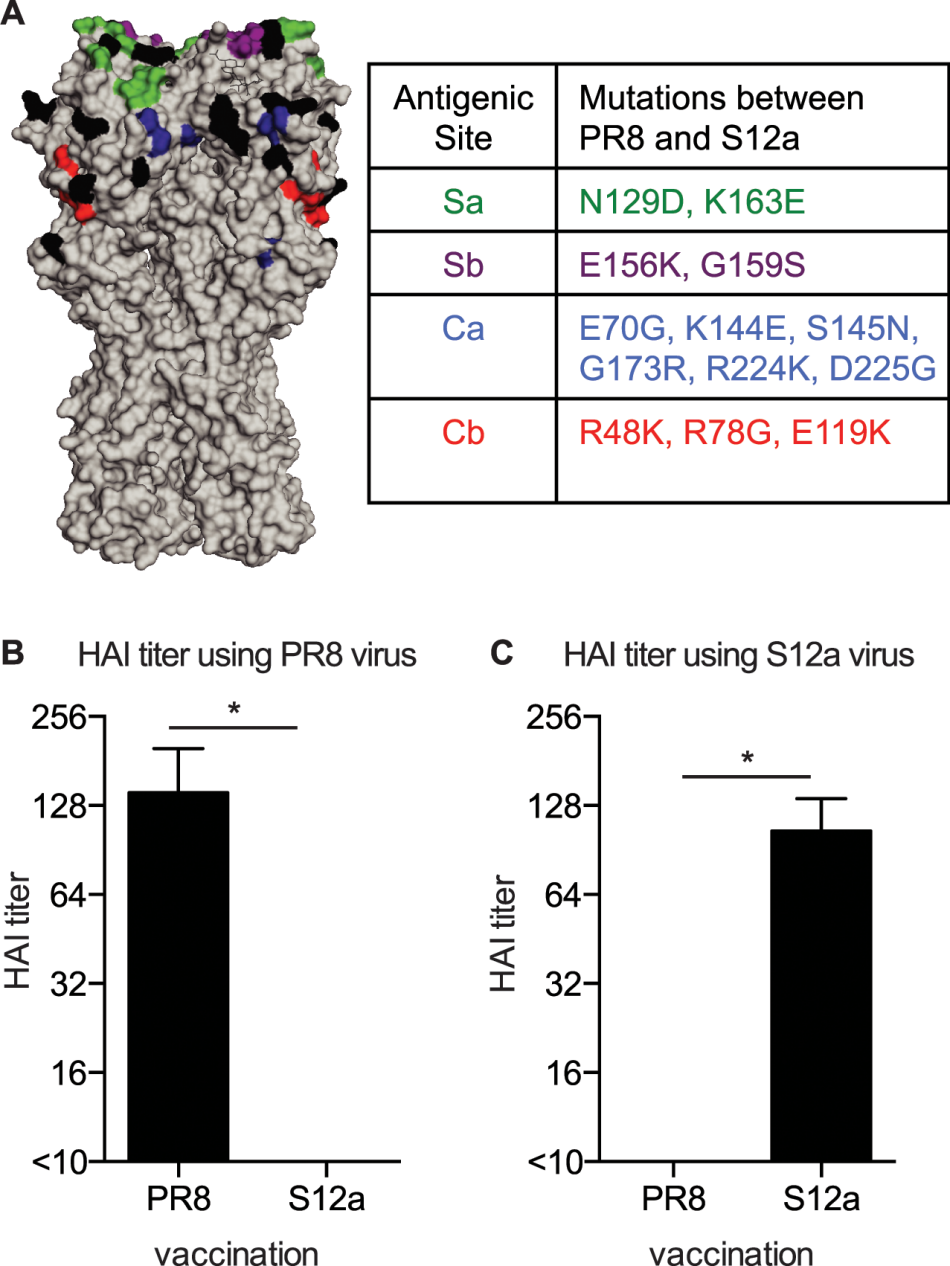
PR8 and S12a are antigenically distinct H1N1 viruses. (A) PR8 and S12a differ at 13 HA residues (highlighted in black) (Protein database: 1RVX). The Sa, Sb, Ca, and Cb antigenic sites are shown in green, purple, blue, and red respectively. (B,C) Mice were immunized i.p. with 1000 HAU PR8 or S12a virus. Sera were collected 21 days post-vaccination and HAI assays were completed using PR8 (B) or S12a (C) virus. Data are mean +/- SD (*p < 0.0001; Student’s t test).

We established a mouse model to determine how prior H1N1 influenza A virus exposures influence the generation of new Ab responses against antigenically drifted H1N1 strains. For these studies, we utilized the well-characterized A/Puerto Rico/8/1934 (PR8) and A/Puerto Rico/8/1934-S12a (S12a) H1N1 strains [1,9,10]. Early monoclonal Ab (mAb) mapping studies demonstrated that there are 4 dominant antigenic sites on the globular head region of the HA of PR8 [1,9]. The HAs of PR8 and S12a differ by 13 mutations spread across the 4 dominant HA head antigenic sites (Fig 1A) [10]. PR8 and S12a viruses are antigenically distinct as determined by hemagglutination-inhibition (HAI) assays (Fig 1B and 1C), which measure Ab inhibition of virus binding to red blood cells. Although the HA stalk region of these viruses is identical (Fig 1A), 98% of PR8-elicited mAbs fail to recognize a virus closely related to the S12a virus in direct binding assays [10].

We exposed BALB/c mice to PR8 virus and then re-exposed the same mice 28 days later with the identical PR8 virus or the antigenically distinct S12a virus. Inactivated viruses were used for these studies to mimic conventional influenza vaccinations. Initial viral exposures were given via the intraperitoneal (i.p.) route and secondary exposures were given via the intravenous (i.v.) route. Secondary exposures were delivered via the i.v. route to enhance our ability to derive hybridomas from sequentially exposed mice.

**Fig 2:**
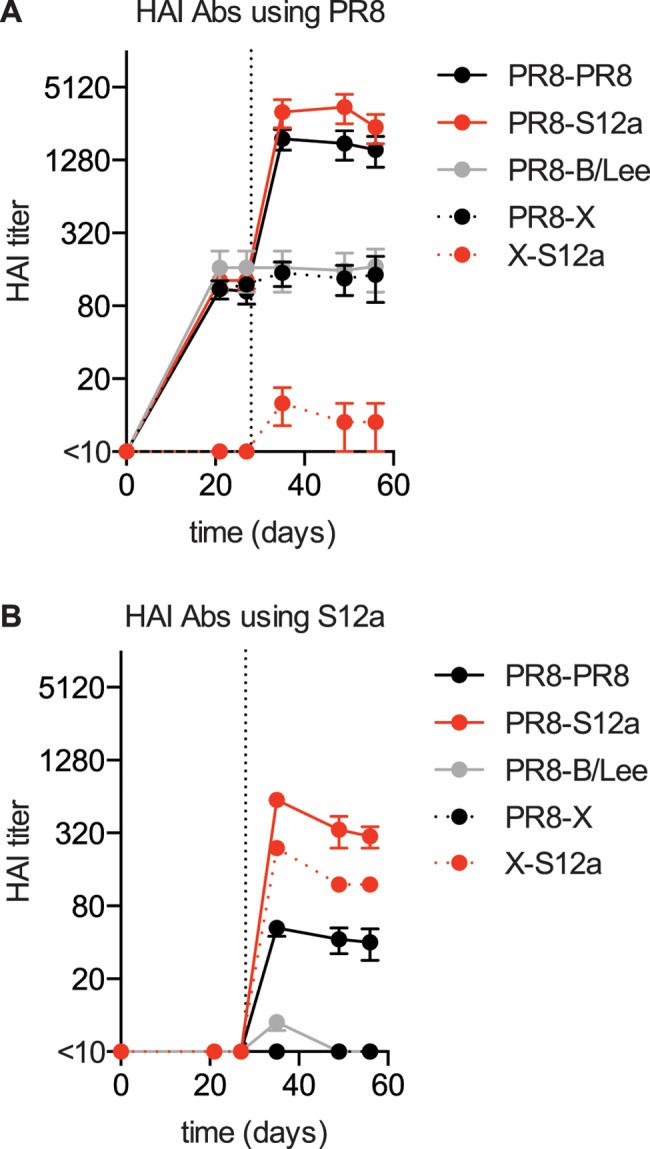
S12a efficiently boosts Abs that react to PR8 HA. (A,B) Mice were immunized i.p. with 1000 HAU of PR8 or PBS (X) and boosted 28 days later i.v. with 1000 HAU PR8, 1000 HAU S12a, 1000 HAU B/Lee, or PBS (X) (n = 4 mice per group). Sera were collected at different times after immunization and HAI assays were completed using PR8 (A) and S12a (B) viruses. Data are mean +/- SEM. Mice boosted with both PR8 or S12a had significantly higher PR8 HAI titers compared with mice boosted with PBS (X) or the antigenically unrelated B/Lee virus (p < 0.05; Student’s t test).

We first measured PR8-reactive Abs in sera collected from these mice. As expected, PR8-reactive Abs were boosted upon re-exposure with PR8 (Fig 2A). Surprisingly, the antigenically distinct S12a virus was also able to boost PR8-reactive Abs to levels comparable to that elicited by a homologous PR8-PR8 prime-boost (Fig 2A). Similar results were obtained when we exposed mice with S12a virus 70 days after the initial PR8 exposure (S1A Fig). Since HAI assays only detect Abs directed against the globular head of HA, we completed additional direct ELISA binding assays. Consistent with our HAI data, PR8-reactive Abs detected by ELISA (which measure head and stalk HA Abs) were similar in sera collected from mice sequentially exposed to PR8 twice and mice sequentially exposed to PR8 and the antigenically distinct S12a virus (S1B Fig). S12a boosting of the PR8 response was not due to non-specific activation of B cell clones initiated by the first viral exposure, since an influenza B strain (B/Lee; unrelated to the PR8 and S12a H1N1 influenza A strains) did not boost PR8 Abs in mice originally exposed to PR8 (Fig 2A). Similarly, a virus expressing an H3 HA (J1 virus) and a recent human H1N1 virus (A/California/7/2009) were unable to boost PR8 HAI titers in mice pre-exposed to PR8 (S1A and S1C Fig).

Classically, OAS describes the process by which B cells stimulated by past viral exposures dominate and suppress the generation of *de novo* activated B cells that recognize new antigens present in drifted viral strains [5]. In mice, classical OAS is observed following sequential infection with antigenically distinct live viruses [7]. It is interesting that the perceived suppression of *de novo* Ab responses following sequential infection is most apparent following live virus infection. Cross-reactive Abs can limit viral replication during secondary viral infections, and this in turn can limit levels of antigen. Jacobs and colleagues showed that the apparent suppression of Ab responses in some OAS studies is likely due to decreased amounts of antigen expressed during secondary infections [7]. To determine if PR8 pre-exposure prevents the generation of *de novo* Ab responses capable of recognizing S12a, we measured levels of S12a-reactive Abs in mice sequentially exposed to inactivated viruses. Similar levels of S12a-reactive Abs were present in S12a exposed mice with or without prior PR8 exposure (Fig 2B).

To determine if S12a-reactive Abs elicited in pre-exposed mice were able to prevent S12a infection, we transferred sera from immunized mice into naïve mice and then challenged these mice with S12a. Consistent with the HAI data (Fig 2B), passively transferred sera elicited by S12a primary exposure and PR8-S12a sequential exposure were able to protect mice against S12a infection (S2 Fig). Taken together, our data suggest that in PR8-exposed mice, inactivated S12a elicits Abs that react to the antigenically distinct PR8 strain but that this does not occur at the expense of eliciting S12a-reactive Abs.

### Identification of Abs with an OAS phenotype

**Fig 3:**
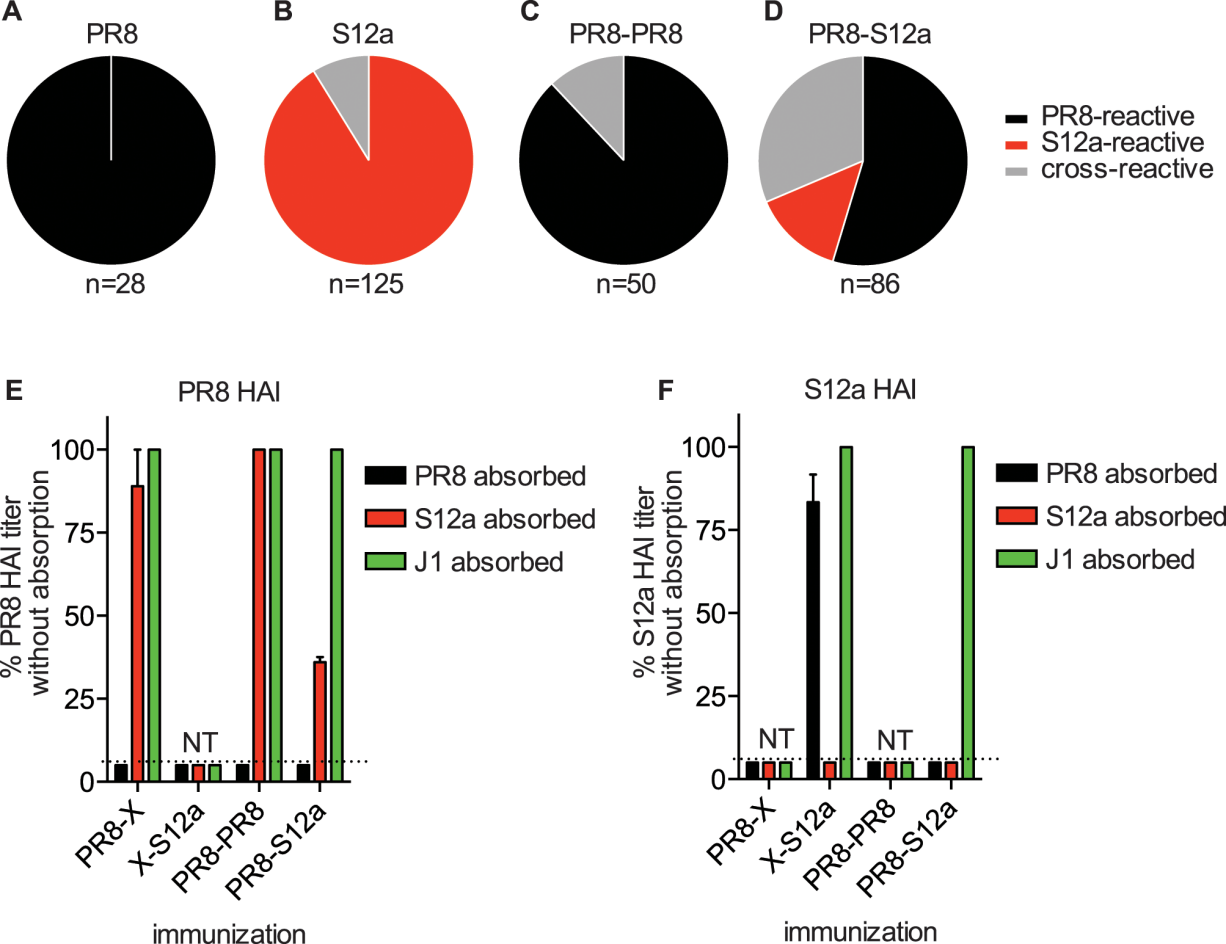
PR8-S12a sequential exposure elicits many Abs with a classical OAS phenotype. (A, B) Mice were immunized i.v. with 1000 HAU of PR8 (A) or S12a (B) virus. Hybridomas were created from splenocytes collected 5 days after vaccination. (C, D) Mice were immunized i.p. with 1000 HAU of PR8 and boosted i.v 28 days later with 1000 HAU PR8 (C) or S12a (D). Hybridomas were produced 3 days after the boost. We completed ELISAs with a range of different mAb concentrations using plates coated with PR8, S12a, and J1 (J1 is a virus that has an H3 HA). mAbs were determined to be PR8 and/or S12a-reactive if they produced an ELISA signal that was 4 times greater using PR8 or S12a coated plates compared to plates coated with the J1 negative control. Mice boosted with S12a had a higher proportion of Abs cross-reactive for both PR8 and S12a (p < 0.005; Fisher’s exact test). (E,F) HAI assays were completed with sera that were pre-incubated with MDCK cells infected with PR8, S12a, or J1 (an H3 negative control). For these experiments, we used sera collected 21 days after the second viral exposure. Data are expressed as % of HAI titer using unabsorbed sera (100% indicates titer was not reduced by absorption). Data are mean +/- SEM; n = 3 mice per group. NT = no titer.

Our experiments using sera cannot distinguish whether cross-reactive Abs are preferentially elicited in sequentially exposed mice or if PR8-specific Abs and S12a-specific Abs are both elicited in sequentially exposed mice. To determine the specificity of Abs elicited in our experimental system, we created a panel of 289 hybridoma cell lines from mice exposed to only PR8, to only S12a, sequentially to PR8 twice, or sequentially to PR8 and S12a (Fig 3). Hybridoma cell lines were created from a total of 23 mice (S3 Fig). Consistent with previous studies [10], a single PR8 exposure elicited monoclonal Abs (mAbs) that bound with a high affinity to PR8 but not S12a (Fig 3A). Likewise, the majority of mAbs elicited by a single S12a exposure bound with a high affinity to S12a but not to PR8 (Fig 3B). Most mAbs elicited by PR8-PR8 sequential exposure bound with a high affinity to PR8 and not S12a, although some mAbs elicited under these conditions were able to bind to both viruses (Fig 3C). Sequential exposure with PR8 and S12a elicited a higher proportion of mAbs that were able to bind with a high affinity to both PR8 and S12a (Fig 3D; p < 0.05; Fisher’s exact test). Almost all of these cross-reactive mAbs bound to PR8 better than to S12a (example binding curves shown in S4 Fig). S12a-specific mAbs were also elicited by PR8-S12a sequential exposure (Fig 3D). Strikingly, approximately ½ of the mAbs elicited by sequential exposure to PR8 and S12a had an ‘OAS’ phenotype since they bound with a high affinity to only PR8 and not S12a (Fig 3D). Most of these ‘OAS’ mAbs bound to S12a so poorly that actual binding affinities to S12a could not be calculated using standard ELISA assays (S5 Fig). As a control, we attempted to create hybridoma cell lines from PR8-exposed mice that were subsequently exposed to an unrelated influenza B strain. We were unable to isolate PR8-reactive mAbs under these conditions, which indicates that some level of antigenic-relatedness is required to recall PR8-reactive Abs in PR8-exposed mice.

The hybridomas in these experiments were created using splenocytes that were isolated 3 days after the second viral exposure. It is possible that this strategy preferentially gives rise to hybridomas that produce Abs that cross-react to PR8 since we isolated splenocytes early after secondary viral exposure. To address this, we quantified the amount of PR8-reactive Abs in polyclonal sera collected at a later time point (21 days) following secondary viral exposure. We quantified PR8- and S12a-reactive Ab titers in sera that were absorbed with cells infected with either PR8, S12a, or J1 (an H3 negative control). In these experiments, PR8-reactive Abs bind PR8-infected cells, and a decrease in Ab titer after absorption with PR8-infected cells indicates the presence of PR8-reactive Abs. Conversely, S12a-reactive Abs bind to S12a-infected cells, and a decrease in Ab titers after absorption with S12a-infected cells indicates the presence of S12a-reactive Abs. Abs that bind to both PR8 and S12a are absorbed by both PR8- and S12a-infected cells in these assays. Consistent with our hybridoma data, PR8 HAI Abs in sera from mice that received a single PR8 exposure or sequential PR8-PR8 exposures were completely absorbed by cells infected with PR8 but not by cells infected with S12a (Fig 3E). Similarly, S12a HAI Abs in sera from mice that received a single S12a exposure were completely absorbed by cells infected with S12a but not by cells infected with PR8 (Fig 3F). Analysis of sera from mice sequentially exposed with PR8 and S12a confirmed the presence of PR8-dominated cross-reactive responses 21 days following S12a exposure. PR8 HAI Abs in sera from mice that were sequentially exposed to PR8 and S12a were completely absorbed with cells infected with PR8 and partially absorbed with cells infected with S12a (Fig 3E). Unlike sera from mice exposed to a single S12a exposure, S12a HAI Abs in sera from mice sequentially exposed to PR8 and S12a were completely absorbed by cells infected with PR8 (Fig 3F). These experiments indicate that PR8-S12a sequential exposure elicits some Abs that only bind efficiently to PR8 and other Abs that are capable of binding to both PR8 and S12a.

### Specificity and functionality of mAbs with an OAS phenotype

**Fig 4:**
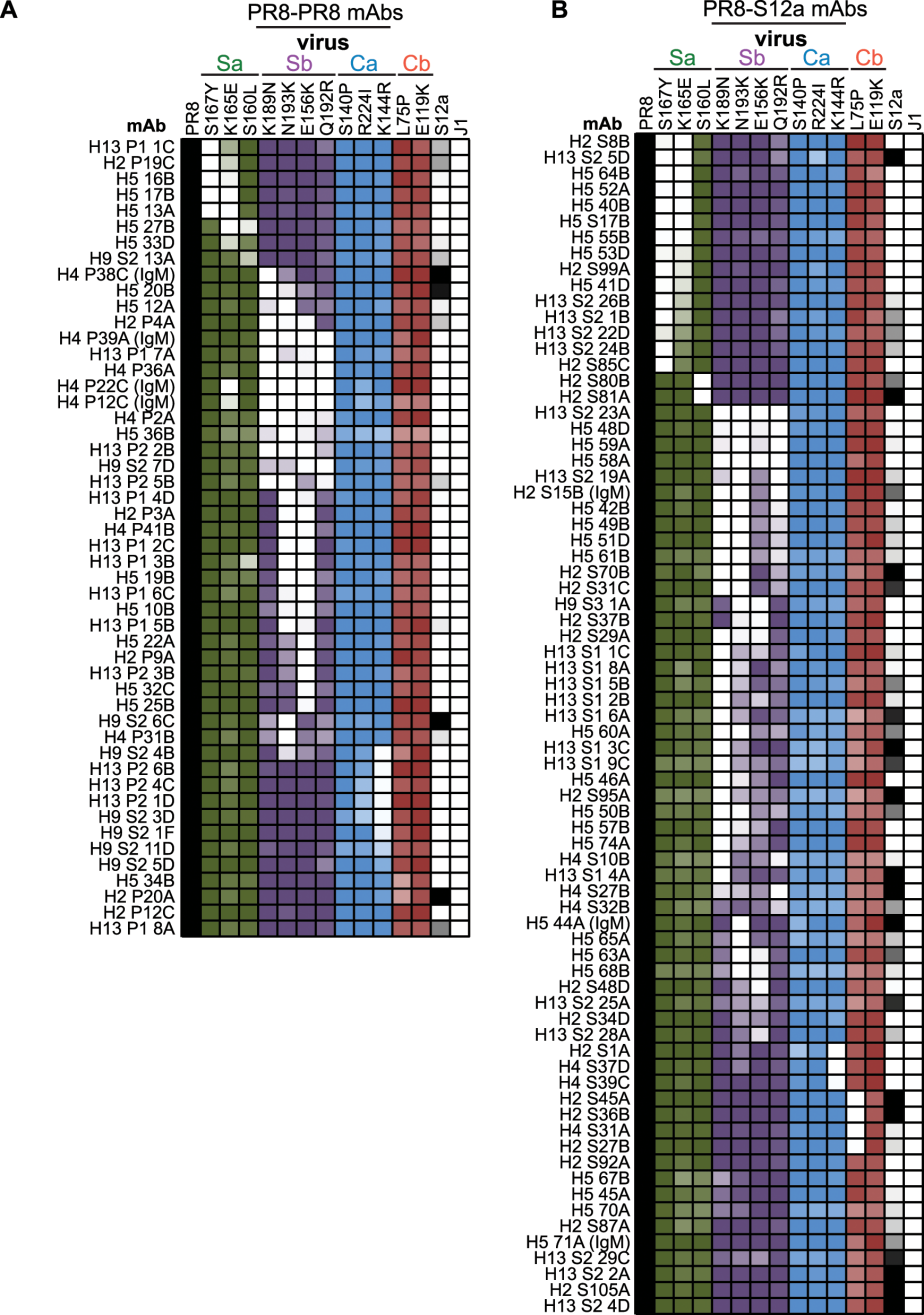
Fine specificity of mAbs elicited by sequential H1N1 exposure. (A, B) mAbs created from PR8 pre-exposed mice that were boosted either with PR8 (A) or S12a (B) were tested for binding to a panel of viruses by ELISA. We first completed a dose titration of each mAb on ELISA plates coated with PR8. We determined the lowest concentration of each mAb at which PR8 ELISA signal was within 90% of Bmax, and we tested this mAb concentration against all of the mutant viruses. The shade of color in each square is relative to the percentage of binding to that virus compared to PR8 binding with the darkest color being 100% and white being 0%. The coloring scheme (Sa = green, Sb = purple, Ca = blue, Cb = red) matches the coloring scheme of the HA structure shown in Fig 1. mAbs are IgG unless otherwise noted.

We mapped the binding footprints of each mAb by measuring binding to a large panel of PR8 viruses that possessed single mutations in the HA (Fig 4). Our mutant PR8 viral panel included previously characterized mAb escape mutants [1], as well as new escape mutants that we isolated after incubating PR8 virus with mAbs from this study (for example, the K189N HA mutant was isolated after growing PR8 virus in the presence of the H5-60A mAb). We tested mAb binding to viruses that possessed single mutations across the 4 dominant HA antigenic sites (Sa, Sb, Ca, and Cb). The majority of mAbs isolated from mice sequentially exposed to PR8-PR8 and PR8-S12a did not bind to PR8 viruses possessing Sb mutations (Fig 4A and 4B and S6 Fig), indicating that both PR8-PR8 and PR8-S12a sequential exposures elicit an Ab response that is immunodominant against epitopes in the Sb antigenic site of HA. Approximately ½ of the Sb mAbs isolated from mice sequentially exposed to PR8 and S12a displayed a classical OAS phenotype since they bound poorly to S12a (Fig 4B). Importantly, the other ~ ½ of the Sb mAbs isolated from mice sequentially exposed to PR8 and S12a were cross-reactive with strong binding to both PR8 and S12a (Fig 4B). mAbs isolated from mice receiving only a single PR8 or S12a exposure reacted to all 4 HA globular head antigenic sites and were not as dominated against the Sb antigenic site (S6 Fig), indicating that sequential viral exposure focused the Ab response towards the Sb antigenic site.

**Fig 5:**
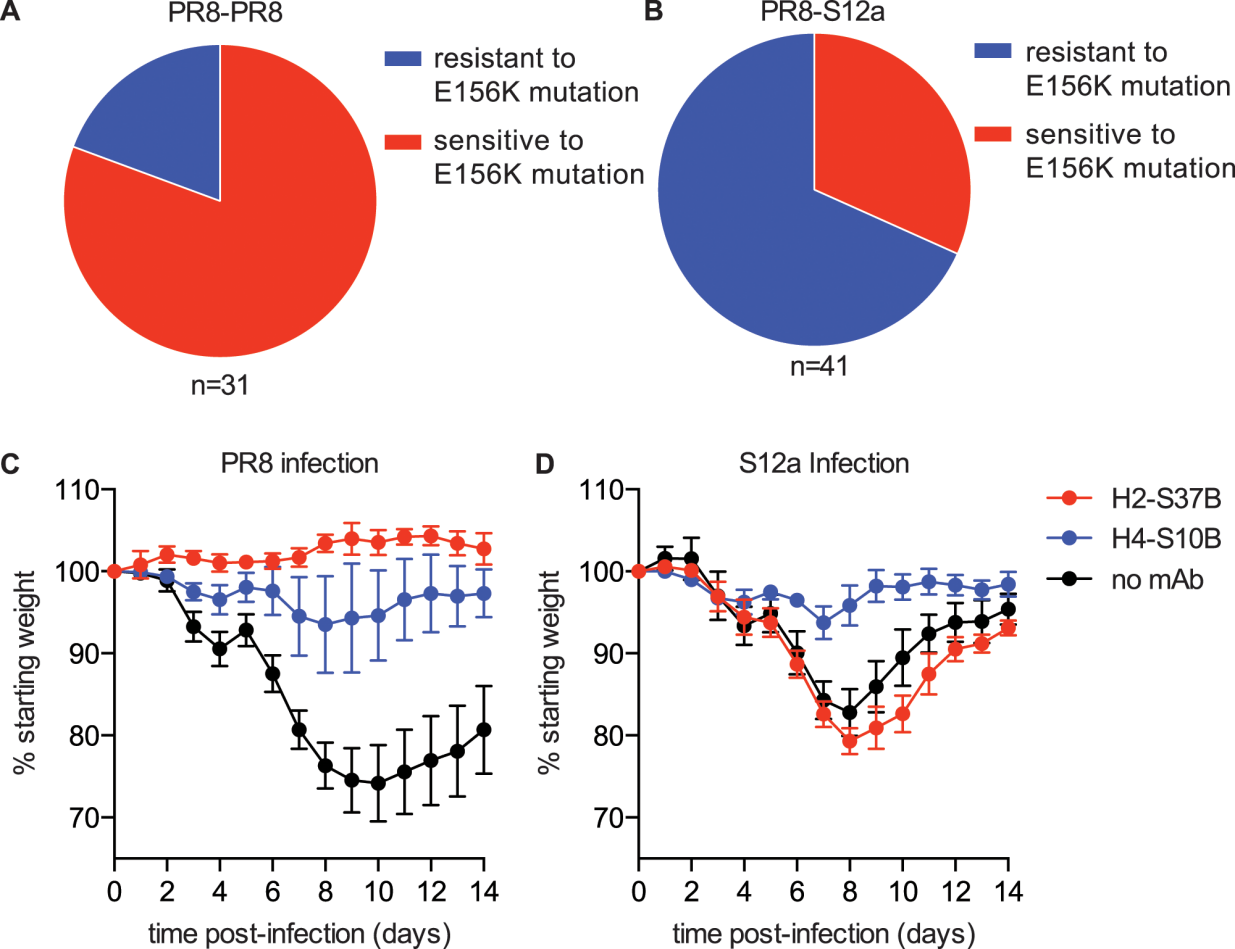
Most Sb mAbs elicited by PR8-S12a sequential exposure are not sensitive to the E156K mutations and protect against PR8 and S12a infection. (A, B) Sb-specific mAbs from mice pre-exposed to PR8 virus and boosted with either PR8 (A) or S12a (B) virus were tested for binding by ELISA to PR8 viruses +/- the E156K HA mutation. mAbs that bound to the E156K mutant >25% relative to PR8 were considered resistant to the E156K mutation. PR8-S12a exposure elicited a higher proportion of mAbs that were resistant to the E156K mutation compared to PR8-PR8 exposure (p < 0.0001; Fisher’s exact test). (C, D) 25ug of the H2-S37B or H4-S10B mAbs were transferred i.p. into mice 12 hours before infection with 30 TCID50 of PR8 (C) or S12a (D) virus. Weight loss was measured for 14 days post-infection. PR8-infected mice that received either mAb lost significantly less weight than mice that received a PBS control (no mAb) (p < 0.05 at days 6–14 for H2-S37B and H4-S10B; two way ANOVA). S12a-infected mice that received the H4-S10B mAb lost significantly less weight than mice that received a PBS control (p < 0.05 at days 6–11; two way ANOVA). Data are mean +/- SEM.

We next sought to determine if there are fine specificity differences that distinguish Sb mAbs isolated from mice exposed with PR8-PR8 compared to PR8-S12a. To do this, we completed additional mAb binding experiments with a larger panel of PR8 viruses with single Sb mutations (S7 Fig). There was a notable key difference in the binding footprint of Sb mAbs isolated from mice sequentially exposed to PR8-PR8 compared to PR8-S12a: the majority (70%) of Sb mAbs isolated from mice sequentially exposed twice with PR8 failed to bind to viruses with a mutation at HA residue 156, whereas the minority (20%) of Sb mAbs isolated from mice sequentially exposed with PR8 and S12a were sensitive to the 156 mutation (Fig 5A and 5B and S7 Fig). This is important because the Sb antigenic site of PR8 and S12a differ at residue 156 (PR8 has E156 and S12a has K156). Most of the ‘OAS’ Sb Abs that were elicited by PR8-S12a sequential exposure did not bind efficiently to PR8 viruses with an E156K HA mutation (these Abs had a similar footprint to most Sb Abs elicited by PR8-PR8 sequential exposure), whereas most of the cross-reactive Sb Abs elicited by PR8-S12a sequential exposure were less affected by the E156K mutation (Fig 4B and S7 Fig).

To determine if mAbs isolated from mice sequentially exposed to PR8 and S12a can protect against viral infection, we completed a series of passive transfer studies in mice. We transferred mAbs that were either sensitive (H2-S37B) or resistant (H4-S10B) to the E156K HA mutation (Fig 4B) and we then challenged mice with either PR8 or S12a virus. Both mAbs protected mice against PR8 infection (Fig 5C). The H2-S37B mAb that was sensitive to the PR8 E156K HA mutation failed to protect mice against the S12a virus, however the H4-S10B mAb that was resistant to the PR8 E156K HA mutation fully protected mice against the S12a virus (Fig 5D). The H4-S10B mAb was able to protect against S12a virus infection, despite binding to S12a with a much lower affinity compared to PR8 (Fig 4B and S8 Fig).

These data suggest that sequential exposure with PR8 and S12a elicit two closely related but clearly distinct types of Sb-specific Abs. The first type of Ab displays a classical OAS phenotype in that these Abs do not efficiently recognize S12a or a PR8 virus engineered to have the E156K HA mutation, which is the mutation that is largely responsible for the Sb antigenic difference between PR8 and S12a. The second type of Ab targets the same general Sb region but binds to both PR8 and S12a and is not as sensitive to variation at residue 156. These cross-reactive Abs appear to have a narrower binding footprint based on our binding assays using a large panel of Sb mutant PR8 viruses (S7 Fig).

### Somatic mutations may contribute to cross-reactivity of Sb mAbs

**Fig 6:**
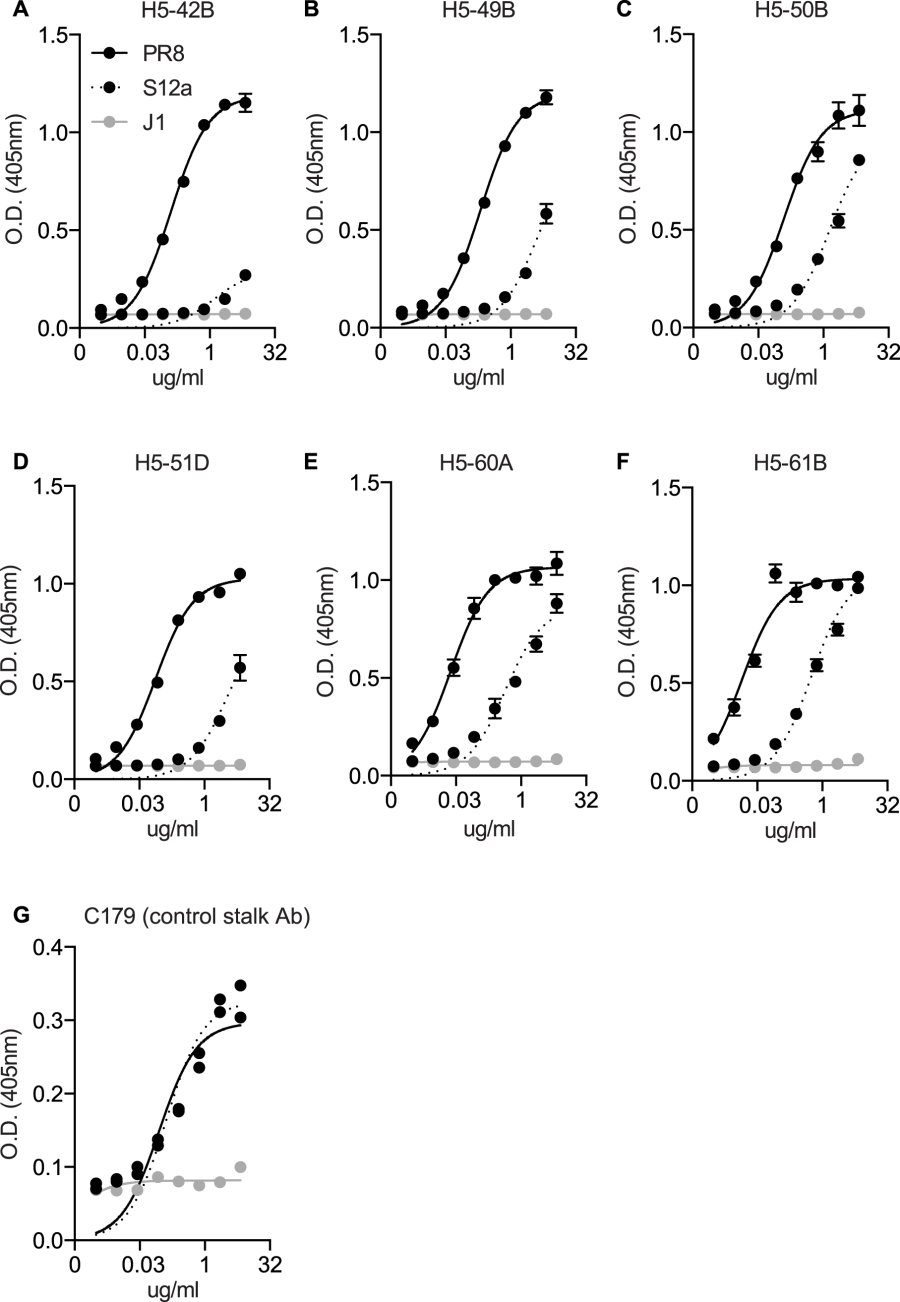
Somatic mutations affect binding to both PR8 and S12a. (A-F) Six clonally related (see S1 Table) IgG2a mAbs derived from a mouse primed with PR8 and boosted with S12a were tested for binding to PR8, S12a, and the antigenically unrelated J1 virus via ELISA (J1 is a virus that has an H3 HA). Data are mean +/- SEM. (G) As an ELISA coating control, the H1 stalk-reactive C179 mAb was tested for binding to PR8, S12a, and J1 virus. Data are mean +/- SEM.

In an attempt to identify clonally-related mAbs, we sequenced the heavy and light chains of a number of our hybridoma cell lines. We identified a group of 6 clonally-related IgG2a mAbs derived from a single mouse that was sequentially exposed to PR8 and S12a (S1 Table). These 6 related mAbs had very different binding affinities to PR8 and S12a (Fig 6). For example, the H5-42B mAb had very weak binding to S12a (Fig 6A), whereas the H5-61B mAb, which differed by only 4 amino acids in the heavy chain relative to H5-42B (1 CDR1, 2 FR3, and 1 CDR3 differences), had dramatically increased binding to S12a (Fig 6F). It is unclear if the mutations that differentiate H5-42B and H5-61B originated during the initial priming with PR8 virus or during the secondary recall response against S12a virus. Regardless, these data suggest that somatic mutations can influence the level of cross-reactivity of Abs elicited by secondary influenza exposures.

**Fig 7:**
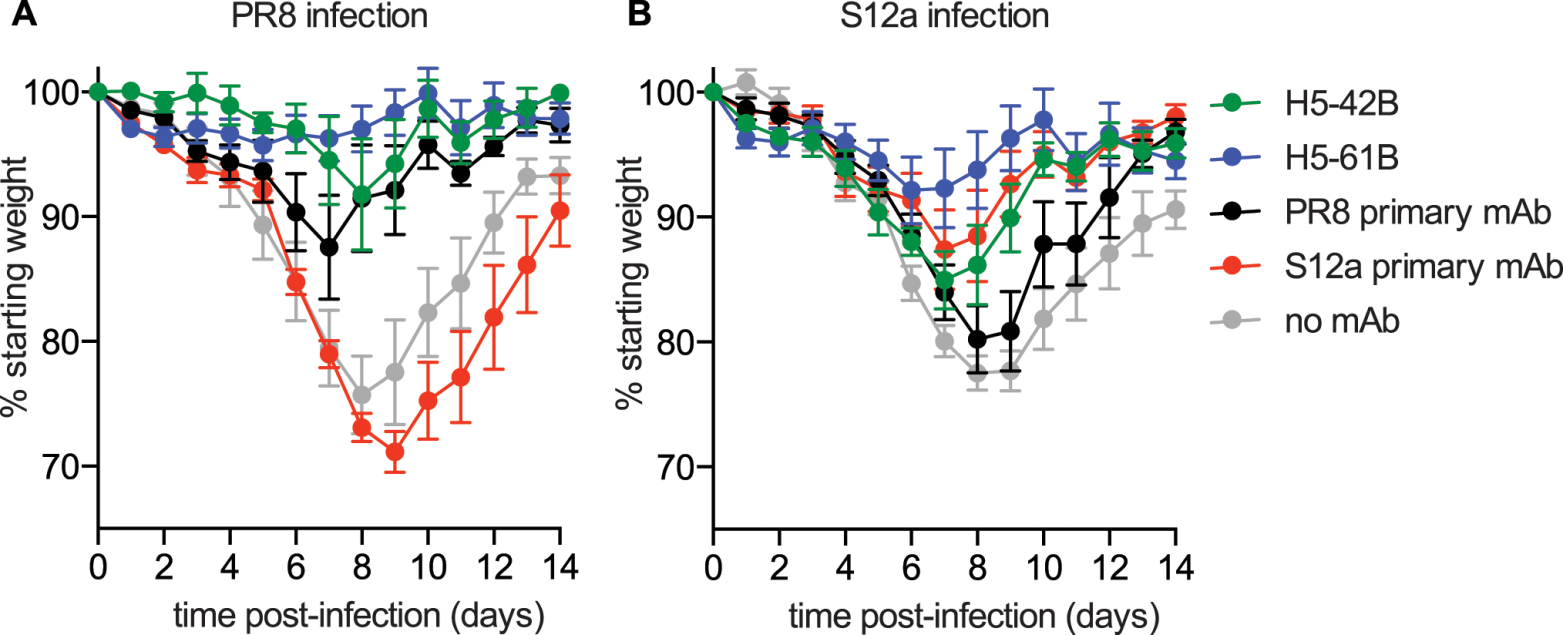
mAbs elicited by PR8-S12a immunization prevent disease caused by PR8 and S12a infections. (A, B) 25ug of different mAbs were transferred i.p. into mice 12 hours before infection with 30 TCID50 PR8 (A) or S12a (B). PR8-infected mice that received H5-42B, H5-61B, or the PR8 primary mAb lost significantly less weight than mice that received a PBS control (no mAb) (p < 0.05 at days 5–12 for H5-42B, p < 0.05 at days 6–12 for H5-61B, p < 0.05 at days 6–11 for the PR8 primary mAb; one way ANOVA). S12a-infected mice that received the H5-42B, H5-61B, or S12a primary mAb lost significantly less weight than mice that did not receive mAbs (p < 0.05 at days 6–12 for H5-42B and H5-61B, p < 0.05 at days 6–14 for S12a primary mAb; one way ANOVA). Data are mean +/- SEM.

To determine if the H5-42B and H5-61B mAbs have different protective efficiencies, we completed *in vivo* passive transfer experiments. Both the H5-42B and H5-61B mAbs protected against PR8 infection following passive transfer *in vivo* (Fig 7A). Surprisingly, despite the observation that H5-42B binds poorly to S12a (Fig 6A), both mAbs also protected against S12a infection following passive transfer *in vivo* (Fig 7B). The H5-61B mAb, which had a higher relative binding affinity to S12a compared to H5-42B, was more protective following S12a infection *in vivo* compared to H5-42B (Fig 7B). As a comparison, we also tested mAbs isolated from mice following a primary PR8 exposure (the H0-P2D5D10 mAb) or a primary S12a exposure (the H7-S2-2A mAb). As expected, the PR8 primary mAb protected mice only against PR8 infection and the S12a primary mAb protected mice only against S12a infection (Fig 7A and 7B). Importantly, the S12a primary mAb protected mice against S12a infection to the same extent as the H5-42B mAb elicited by PR8-S12a sequential exposure. These data indicate that Abs elicited by sequential exposures can be highly effective at protecting against the virus that recalls them, even though they often bind with a much higher affinity to the first viral strain that the host encountered. These Abs can be as effective as Abs elicited in a previously naïve host.

## Discussion

Most vaccines elicit protective Abs and memory B cells that are poised to respond quickly to a secondary exposure [11]. Formulating effective vaccines against rapidly changing pathogens, such as influenza viruses, is challenging [12–14]. Influenza vaccines are less effective when viruses acquire mutations in Ab binding sites on the HA, as was the case during the 2014–2015 influenza season [15]. B cells primed against older influenza strains are recruited upon exposure with new influenza strains, even when major HA antigenic changes have occurred. For example, prior seasonal H1N1 exposure heavily influenced the types of Abs that were elicited in humans against the 2009 pandemic H1N1 strain [16–19]. mAbs isolated from humans exposed to the 2009 pandemic H1N1 strain recognize epitopes that are conserved on older seasonal H1N1 strains, and these mAbs have a high level of somatic mutation indicating that they were likely derived from a memory B cell pool [19]. Immune skewing towards epitopes present in older viral strains is likely a result of memory B cells clones outcompeting naïve B cell clones that are specific for epitopes present in new viral strains [20–22]. In the case of the 2009 pandemic H1N1 strain, many of these cross-reactive mAbs were protective and so the recall of B cell clones originally primed by seasonal H1N1 viruses was likely advantageous to the host.

There is evidence that prior influenza exposures can negatively affect priming of Ab responses against new viral strains through a process commonly called OAS [5,23–26]. Prior influenza exposures can decrease the generation of *de novo* Ab responses, but this is partially due to cross-reactive Abs (originally elicited by prior exposures) binding to and eliminating antigen following secondary exposure with antigenically drifted strains [7]. New *de novo* Ab responses are not primed effectively when cross-reactive Abs limit antigen levels. In this context, prior immunity is again advantageous because Abs elicited against prior influenza exposures are partially protective and limit the amount of virus and antigen expressed upon secondary exposure.

A poorly understood component of OAS is that antigenically distinct viruses recall some Abs that paradoxically only react to older viral strains and not to the viral strain that is actually eliciting the response. Our data indicate that there are surprisingly only subtle differences between Abs with an OAS phenotype and cross-reactive Abs. We expected that sequential exposure with the PR8 and S12a strains would elicit Ab responses directed towards epitopes that are fully conserved between the two viral strains. However, we found that the majority of Abs elicited by PR8-S12a sequential exposure were directed against the Sb antigenic site of HA, which differs significantly between the two viral strains. Some Sb-specific mAbs elicited by sequential exposure exhibited a classical OAS phenotype in that they bound poorly to S12a but bound with a high affinity to PR8. Other Sb-specific mAbs elicited in the same mice were more cross-reactive and bound to both PR8 and S12a. It is unclear if OAS and cross-reactive Abs recognize distinct epitopes within the Sb antigenic site, or if the cross-reactive Sb Abs were simply more tolerant of differences within this antigenic site. Very few somatic mutations were required to increase S12a-reactivity of OAS Abs, indicating that B cells expressing OAS and cross-reactive Abs can be clonally related.

It is difficult to identify precise viral exposure histories in humans. The murine studies presented in this manuscript allowed us to carefully study how specific viral exposures influence Ab responses against antigenically drifted influenza strains. However, there are some limitations of our murine system. For example, most humans have been exposed to multiple different influenza strains (not just two viral strains) and Abs of different specificities might be more prevalent in older humans versus younger humans. Nonetheless, our murine studies have provided important insights into how prior influenza virus exposures shape Ab responses elicited against new viral strains.

Although OAS Abs are typically thought of as detrimental, Abs with an OAS phenotype elicited in our experiments were able to protect mice from S12a infections in passive mAb transfer experiments. These Abs were able to protect mice as efficiently as Abs generated during a primary immune response. Although Abs with an OAS phenotype bind to virus with a very low affinity, our data suggest that this binding is sufficient for limiting viral replication. It is possible that Abs with an OAS phenotype protect via other mechanisms, such as increasing antigen uptake and immune complex formation. We propose that Abs classically associated with OAS might play an important role in protecting the host against secondary encounters with antigenically drifted viral strains.

## Materials and Methods

### Ethics statement

BALB/c mice (Charles River Laboratories) were used for all experiments. All murine experiments were performed at the Wistar Institute (OLAW # A3432-01) according to protocol #112338 approved by the Wistar Institute Institutional Animal Care and Use Committee. This protocol adheres to the National Institutes of Health’s *Public Health Service Policy on Humane Care and Use of Laboratory Animals*.

### Viruses

Viruses were grown in 10-day old fertilized chicken eggs and the HA genes of each virus stock were sequenced to verify that additional mutations did not arise during propagation. S12a has an additional K144E HA mutation compared to the previously published SEQ-12 virus [10]. S12 viruses possessing the K144E HA mutation grow efficiently in fertilized chicken eggs without acquiring additional HA mutations.

### HAU assays

To determine the hemagglutination unit (HAU) titer of each viral prep, viruses were diluted in a total volume of 50ul 2-fold across a 96-well round bottom plate (BD) and mixed with 12.5ul of 2% (vol/vol) turkey erythrocytes (Lampire) in PBS. Agglutination was read out after incubating for 60 minutes at room temperature.

### TCID50 assays

96-well flat bottom plates (BD) were incubated overnight with 4e4 MDCK cells (source: the National Institutes of Health) per well in MEM with 9% FBS. Plates were washed 3 times with serum-free MEM. Viruses were diluted 10-fold across the plate in MEM supplemented with 0.1% gentamicin (Gibco), 1ug/ml TPCK-treated trypsin (Worthington), and 5 mM HEPES (Cellgro).

### HAI assays

Sera were collected from mice by submandibular bleeding into 1.1ml Serum Gel Z microtubes (Sarstedt) using a 5 mm lancet (Medipoint). Sera were then heat treated for 30 minutes at 55C. HAI titrations were performed in 96-well round bottom plates (BD). Sera were serially diluted 2-fold in PBS and added to 4 agglutinating doses of virus in a total volume of 100 μL. Turkey erythrocytes (Lampire) were added [12.5 μL of a 2% (vol/vol) solution]. The erythrocytes were gently mixed with sera and virus and agglutination was read out after incubating for 60 min at room temperature. HAI titers are expressed as the inverse of the highest dilution that inhibited 4 agglutinating doses of virus.

### Absorption assays

MDCK cells were infected with 1e7 TCID50/ml PR8, S12a, or J1 virus in MEM supplemented with 0.1% gentamicin, 1ug/ml TPCK-treated trypsin, and 5 mM HEPES. The next day, the infected cells were removed from the flask with 20 minute treatment with 0.25% Trypsin with 2.21mM EDTA (Corning), and washed 4 times with MEM with 9% FBS. The infected MDCK cells were spun down at 300 RCF for 3 minutes and resuspended with sera isolated from immunized mice. For this, mouse serum was diluted 1:40 in 1% BSA in PBS. Sera were incubated with cells for one hour at 4C. To remove residual virus that could interfere with subsequent HAI assays, we then incubated sera with packed turkey red blood cells for 30 minutes at 4C. We centrifuged at 800 RCF for 3 minutes and then incubated with new packed turkey red blood cells for an additional 30 minutes at 4C. We centrifuged at 800 RCF for 3 minutes and then completed HAI assays with absorbed sera.

### mAb ELISAs

Viruses for ELISAs were inactivated by B-Propiolactone (BPL; Sigma Aldrich) treatment. Viruses were incubated with 0.1% BPL and 0.1 M HEPES (Cellgro) overnight at 4C followed by a 90 minute incubation at 37C. 96-well Immulon 4HBX flat-bottom microtiter plates (Fisher Scientific) were coated with 20 HAU per well BPL-treated virus overnight at 4C. Plates were then incubated with 150ul 3% bovine serum albumin (BSA Sigma) in PBS (Cellgro) for 2 hours at room temperature. For initial hybridoma screening, hybridoma supernatants were diluted 1:5 and then added to ELISA plates. For antibody footprint mapping, purified mAbs were added at a starting concentration of 1 to 10 ug/ml and diluted 2-fold across the plate. As a control, we used the stalk-reactive C179 mAb (Clontech). mAbs were incubated on the plates for 2 hours at room temperature. mAb binding was measured by a secondary goat anti mouse IgG or IgM antibody conjugated to alkaline phosphatase (Southern Biotech) incubated for 1 hour at room temperature. Plates were developed with 100ul/well of a 2.7mM PNPP (Thermo Scientific) 0.1M NaHCO3 (Sigma) 1mM MgCl2 (Fisher Scientific) pH 9.8 solution. Absorbance was measured at 405nm. Plates were washed with distilled water between each incubation step.

### Hybridoma production

Some mice were first primed with 1000 HAU of virus with an i.p. injection and boosted 28 days later with 1000 HAU of virus with an i.v. injection. 3 days later, splenocytes from these mice were fused with SP2/0 cells using Polyethylene glycol (Hybrimax 50% (w/v) Sigma). Some mice were primed only once i.v. with 1000 HAU of virus and splenocytes from these mice were fused 5 days later. Fused cells were incubated in 5.7uM azaserine and 100uM Azaserine-hypoxanthine (Sigma) with 10% FBS (Sigma) in DMEM (Cellgro). 10 days later hybridomas were screened by ELISA as described above. Positive clones were subcloned by limiting dilution, and then grown in DMEM supplemented with Zap hybridoma (InVitria). mAbs were purified using PureProteome A/G coated magnetic beads (Millipore). For mAb transfer experiments, monoclonal antibodies underwent buffer exchange into PBS using Zeba desalt spin columns (Thermo Scientific).

### mAb transfer experiments

25 ug of purified mAbs diluted in PBS were transferred into female BALB/c mice (Charles River Laboratories) in a 200ul i.p. injection. 12 hours later, mice were challenged intranasally with a dose of 30 TCID50 of either PR8 or S12a in 50ul PBS. For intranasal infections, mice were first anesthetized by inhalation of isoflurane (Henry Schein). Weight was measured daily for 14 days.

### Immunoglobulin sequencing

DNA was isolated from hybridoma clones using a Gentra Puregene cell kit (Qiagen). Immunoglobulin heavy and light chain genes were amplified by PCR using a Platinum Taq DNA Polymerase High Fidelity kit (Thermo Fisher Scientific) using previously described primers [27,28]. Amplified DNA was run on a 1% agarose (Lonza) in TAE buffer (Corning) gel and DNA was purified from the gel using a Zymoclean gel DNA recovery kit (Zymo Research). DNA fragments were then sequenced by Sanger sequencing. For hybridomas whose productive and non-productive rearrangements were not easily separated by gel electrophoresis, the DNA was subcloned using a TOPO TA Cloning kit with the pCRII-TOPO vector (Invitrogen).

### Statistical analyses

Student’s t tests, ANOVAs, Fisher’s exact tests, and area under the curve analyses were calculated using Graphpad Prism6 software (Graphpad Software Inc).

## Supporting Information

S1 Table Identification of a clonally related family of mAbs isolated from a single mouse. Mutations that differ each mAb in the framework regions (FR) or complementarity-determining regions (CDR) from the heavy chain germline sequence (IGHV5-4*02, IGHD2-14*01, IGHJ4*01) are shown using IMGT numbering. FR1 was not determined because the sequencing primer bound to this region. We also sequenced the CDR3 region of the light chain (IGKV8-28*01, IGKJ5*01) which was identical between all 6 mAbs.
(DOC)

S1 Fig S12a boosts PR8 Abs. (A) Mice were immunized i.p. with 1000 HAU of PR8 or PBS (X). 70 days later, mice were boosted i.v. with 1000 HAU PR8, S12a, J1, or PBS (X). Sera were collected at different times after immunization and HAI assays were completed using PR8 virus. Mice boosted with PR8 or S12a had significantly higher PR8 HAI titers compared to mice boosted with PBS or the unrelated J1 for at least 3 weeks post-boost (p < 0.05; Student’s t test). (B) Mice were immunized i.p. with 1000 HAU PR8 or PBS (X). Mice were boosted i.v. with 1000 HAU PR8, S12a, or B/Lee 28 days later. Sera were collected 21 days after the boost and tested by ELISA against PR8 virus. Sera from mice that were boosted with PR8 or S12a had significantly higher PR8 titers than mice that were boosted with negative controls PBS or B/Lee (p < 0.05 Student’s t test of area under the curve). (C) Mice were immunized i.p. with 1000 HAU PR8 and then boosted i.v. with 1000 HAU PR8, S12a, J1, or A/California/7/2009 (CAL/09). Sera were collected 14 days later and HAI assays were completed with PR8. Data are mean +/- SEM. N = 4 mice per group. (* p < 0.05 Student’s t test; ns = not significantly different)
(EPS)

S2 Fig S12a elicits protective Abs in mice pre-exposed to PR8. Sera were collected from naive mice, mice immunized with only S12a, mice immunized sequentially with PR8 and S12a, and mice immunized sequentially with PR8 and B/Lee. Sera were collected 28 days after the last viral exposure. Sera were passively transferred into naive mice (25ul sera per mouse). 12 hours later, these mice were infected with 30 TCID50 S12a and weight loss was measured for 14 days. Mice that received sera from S12a and PR8-S12a immunized mice lost significantly less weight from days 6–14 post-infection compared to mice that received sera from naive or PR8-B/Lee immunized mice (p<0.05; Two way ANOVA). Shown are mean and SEM. n = 4 animals/group. Data are representative of 2 independent experiments.
(EPS)

S3 Fig Hybridomas for these experiments were derived from many mice. Shown are the fraction of mAbs that were derived from individual mice. (A) 28 mAbs were isolated from mice exposed to PR8. (B) 125 mAbs were isolated from mice exposed to S12a. (C) 50 mAbs were isolated from mice sequentially exposed to PR8 twice. (D) 86 mAbs were isolated from mice sequentially exposed to PR8 and S12a. We did not use many mice for PR8 primary exposure since previous studies have characterized mAbs isolated from mice following a single PR8 exposure.
(EPS)

S4 Fig Most ‘cross-reactive’ mAbs bind better to PR8 compared to S12a. mAbs were tested by ELISA for reactivity against PR8, S12a, and J1 virus. (A-C) Shown are 3 mAbs that bound with a higher relative affinity to PR8 than to S12a. (D) As an ELISA coating control, we used the stalk-specific C179 mAb that bound with similar affinities to both PR8 and S12a.
(EPS)

S5 Fig ‘OAS’ mAbs bind very poorly to S12a. (A-C) mAbs were tested by ELISA for reactivity against PR8, S12a, and J1 virus. Shown are three mAbs elicited in mice that received a PR8-S12a prime-boost vaccination that had measurable binding to PR8 but not to S12a. As an ELISA coating control, we used the stalk-specific C179 mAb that bound with similar affinities to both PR8 and S12a (example shown in S4D Fig).
(EPS)

S6 Fig The HA Ab response is focused on the Sb antigenic site following sequential exposure. (A,B) Hybridomas derived from mice that were vaccinated with PR8 virus and boosted 28 days later with either PR8 (A) or S12a (B) were predominantly specific for the Sb antigenic site. (C,D)This was in contrast to hybridomas derived from mice that were immunized only with PR8 (C) or S12a (D) (p < 0.05; Fisher’s exact test).
(EPS)

S7 Fig Fine mapping of Sb mAbs reveals that most cross-reactive mAbs are not sensitive to an E156K HA mutation. (A, B) mAbs that were derived from mice immunized with PR8 and then boosted 28 days later with either PR8 (A) or S12a (B) were tested for binding to a panel of viruses. Twelve of these viruses had single point mutations at residues in and around the previously defined Sb site. Most S12a-reactive mAbs that were elicited by PR8-S12a sequential exposure were resistant to an E156K HA mutation (highlighted in red). Abs of this phenotype were more prevalent in PR8-S12a exposed mice compared to PR8-PR8 exposed mice (highlighted in red). (C, D) The locations of the point mutations tested in S7A and S7B Fig are highlighted in green on the HA structure shown from the side (C) and from the top (D). Residue 156 is highlighted in red.
(EPS)

S8 Fig mAbs passively transferred in Fig 5 bind poorly to S12a. (A-B) mAbs were tested by ELISA for reactivity against PR8, S12a, and J1 virus. Shown are mAbs used in passive transfer experiments in Fig 5. As an ELISA coating control, we used the stalk-specific C179 mAb that bound with similar affinities to both PR8 and S12a (example shown in S4D Fig).
(EPS)
