# Supplementary material for: Antibodies with ‘Original Antigenic Sin’ Properties Are Valuable Components of Secondary Immune Responses to Influenza Viruses
Source: PLoS Pathog. 2016 Aug 18;12(8):e1005806. doi: 10.1371/journal.ppat.1005806 (PMC4990287; doi:10.1371/journal.ppat.1005806)
Supplement: S1 Table — Mutations that differ each mAb in the framework regions (FR) or complementarity-determining regions (CDR) from the heavy chain germline sequence (IGHV5-4*02, IGHD2-14*01, IGHJ4*01) are shown using IMGT numbering. FR1 was not determined because the sequencing primer bound to this region. We also sequenced the CDR3 region of the light chain (IGKV8-28*01, IGKJ5*01) which was identical between all 6 mAbs. (DOC) [file ppat.1005806.s001.doc]

| mAb | CDR1 | FR2 | CDR2 | FR3 | CDR3 |
| --- | --- | --- | --- | --- | --- |
| H5-61B | a110t (Y37F) | g147a (silent) |  | t196c (Y66H),  a214c (K72Q),  a251g (K84R) | c314t (A105V),  a318g (silent),  g319t (silent) |
| H5-42B |  |  |  | a214c (K72Q),  a251g (K84R),  a263t (Y88F) | g313t (A105S),  a318g (silent),  g319t (silent) |
| H5-49B |  |  |  | a214c (K72Q),  a251g (K84R) | c314t (A105V),  a318g (silent),  g319t (silent) |
| H5-51D | a85t (T29S), a110t (Y37F) |  |  | c198t (silent),  a214c (K72Q),  a251g (K84R) | a318g (silent),  g319t (silent) |
| H5-50B | t105c (silent), a110t (Y37),  c114t (silent) | g147a (silent) | g188c (S63T) | t196c (Y66H),  a214c (K72Q),  a225c (silent),  a251g (K84R),  a253g (N85G), a254g (N85G), g275c (S92T) | a318g (silent),  g319t (silent) |
| H5-60A | t105c (silent), a110t (Y37F) | g147a (silent) |  | t196c (Y66H),  a214c (K72Q),  a251g (K84R) | a318g (silent),  g319t (silent) |
